# The Postprandial Effects of a Moderately High-Fat Meal on Lipid Profiles and Vascular Inflammation in Alzheimer’s Disease Patients: A Pilot Study

**DOI:** 10.4172/2329-9126.1000186

**Published:** 2014-11-27

**Authors:** Robin Altman, Alison H. Keenan, John W. Newman, John C. Rutledge

**Affiliations:** 1 Division of Cardiovascular Medicine, Department of Internal Medicine, School of Medicine, University of California at Davis, Davis, CA 95616, California, USA; 2 Department of Nutrition, University of California at Davis, Davis, CA 95616, California, USA; 3 USDA ARS Western Human Nutrition Research Center, University of California at Davis, Davis, CA 95616, California, USA

**Keywords:** Alzheimer’s disease, Postprandial, Lipid, Oxylipin, Cytokine, Monocyte activation, Vascular inflammation

## Abstract

**Objective:**

Alzheimer’s disease (AD) is a neurodegenerative disease of aging with unknown causative factors. Accumulating evidence suggests that inflammation and neurovascular dysfunction play important roles in AD. The postprandial period following a moderately high-fat meal is associated with vascular inflammation in young, healthy individuals; however, this relationship has not been investigated in Alzheimer’s patients despite their exaggerated inflammatory state.

**Methods:**

Patients with AD and age-matched control subjects were recruited through the UC Davis Alzheimer’s Disease Center. All subjects consumed a moderately high-fat breakfast meal. Fasting and postprandial blood samples were collected for lipid, lipoprotein, and oxylipin analyses, as well as assays for cytokine levels and monocyte activation.

**Results:**

The plasma lipid analyses revealed similar levels of triglycerides and esterified oxylipins between groups, but there was an interaction between postprandial non-esterified fatty acid (NEFA) levels and body mass index in the AD group compared to the control subjects. The AD group also had increased behenic acid and decreased linoleic and oleic acids in the postprandial period; however, these were not significantly different. Inflammatory assays revealed elevated fasting levels of interleukin (IL)-10 and IL-12 p70, but no change in monocyte activation in the AD group.

**Conclusion:**

The postprandial period following a moderately high-fat meal is not associated with an exaggerated inflammatory state in Alzheimer’s patients, and basal esterified oxylipin profiles do not indicate elevated oxidative stress. However, the baseline inflammatory state during fasting in AD patients includes elevated levels of plasma IL-10 and IL-12 p70, which may indicate a balance between immune responses mediated by these interleukins.

## Introduction

Alzheimer’s disease is the most common form of dementia and a rapidly growing health concern worldwide. A growing body of evidence suggests Alzheimer’s disease (AD) is a complex neurological disorder of aging that is influenced by a host of genetic and environmental factors. Epidemiological studies indicate that a high-fat diet may be one of these risk factors, and many studies have investigated the effects of long-term dietary interventions on the prevention of AD [1–6]. The acute postprandial effects of a moderately high-fat meal on potential risk factors for AD, such as the blood lipid profile and vascular inflammation, have been less well-studied.

Alzheimer’s disease is associated with an exacerbated inflammatory state in the brain [7–11], and it has been suggested peripheral vascular inflammation may contribute to the disease. Previous work has shown that acute systemic inflammation appears to exacerbate symptoms of chronic neurodegenerative disease [12], and epidemiological studies demonstrate a possible association between serum inflammatory markers and risk of developing AD or cognitive impairment [13]. Additionally, AD has been strongly linked to the metabolic syndrome, which includes perturbations in the normal blood lipid profile such as elevated cholesterol and triglycerides [14–17]. Although controversy still surrounds the role of triglyceride-rich lipoproteins in AD, most evidence points toward a relationship between elevated plasma triglyceride levels and Alzheimer’s disease [14–18].

In young, healthy individuals, increased vascular inflammation during the postprandial period following a moderately high-fat meal correlates with the peak increase in plasma triglycerides [19–21], and non-esterified fatty acids (NEFAs) serve as important mediators of this inflammatory effect [22,23]. Postprandial vascular inflammation also includes increased platelet and monocyte activation and aggregation, and elevated levels of soluble inflammatory markers such as IL-1β and TNF-α [19]. Additionally, elevated plasma esterified oxylipins indicate inflammatory oxidative stress processes, and may also play an important role in the development and progression of Alzheimer’s disease [24–26].

Inflammation of the cerebrovasculature due to elevated blood lipid levels during the postprandial period may promote neurovascular disease, suggesting a potential mechanism contributing to the brain injury characteristic of AD. The goal of this pilot study was to help elucidate whether the postprandial response following a moderately high-fat meal is associated with a perturbed lipid profile and exaggerated vascular inflammation in Alzheimer’s patients.

## Materials and Methods

### Materials

FACS Lysing Solution, FACS Permeabilizing Solution, and fluorescently-labeled antibodies were obtained from BD Biosciences (San Jose, CA). All Vacutainer blood collection tubes were purchased from BD (Franklin Lakes, NJ). Brefeldin A, sodium azide, and bovine serum albumin (BSA) were obtained from Sigma (St. Louis, MO). Paraformaldehyde was obtained from Polysciences, Inc. (Warrington, PA).

### Subjects

The study protocol was approved by the Institutional Review Board of the University of California, Davis. Seven subjects with probable AD and nine cognitively normal control subjects were recruited through the UC Davis Alzheimer’s Disease Center cohorts. Written informed consent was obtained for all subjects using a consent document and procedure approved by the UC Davis Institutional Review Board. A Capacity Assessment Record was completed for each AD subject to ensure their ability to provide informed consent, and if an individual was not able to provide their own consent, a surrogate consent procedure (including written consent document) was also approved for use by the Institutional Review Board.

All subjects underwent a thorough clinical assessment through the UC Davis Alzheimer’s Center prior to enrollment in the study. The diagnosis of dementia was based on DSM-IV criteria 6, and the diagnosis of probable AD was based on NINCDS-ADRDA criteria. Severity of dementia was determined by the Folstein Mini Mental State Exam (MMSE) [27], and Alzheimer’s patients with a score of 16–26 (inclusive), indicative of mild to moderate dementia, were eligible to participate in the study. Subjects did not have evidence of any of the following: dementia due to another cause, seizure disorder, acquired immune deficiency syndrome, drug or alcohol abuse, current or past (within the past two years) myocardial infarction or congestive heart failure, clinically significant or uncontrolled medical condition, or use of an investigational drug before or during the study. Prior to the study day, subjects discontinued their use of lipid lowering drugs (for thirty days) and aspirin (for 14 days), where applicable. Subjects were matched between groups based on age and body mass index (BMI).

### Anthropometric measurements

Body weight and height were measured on the day of the study by trained personnel using standard practices. BMI was calculated using the standard equation: (body mass in kilograms)/((height in meters)^2^).

### Food diary and food frequency questionnaire

For 72 hours prior to the study day, subjects were asked to complete a food diary that was provided by the study personnel. Subjects recorded food and beverage intake, vitamin and supplement intake, and duration/type of physical activity. For those subjects who were unable to independently complete the food diary, family members and/or caretakers assisted with completion of the diary. On the study day, subjects were asked to complete the Block Brief 2000 Food Frequency Questionnaire (FFQ) (NutritionQuest, Berkeley, CA). The FFQ was completed with the assistance of trained study personnel or a registered research dietician, and when possible, a family member and/or caretaker provided collateral history to help complete the FFQ. Information obtained from the food diaries and FFQs was analyzed to establish the baseline nutritive status of the study subjects, and to identify any abnormalities in their dietary behaviors that could have adversely affected the experimental outcomes. There were no indications that the baseline dietary habits of the subjects would have significantly affected the outcomes of the study.

### Study protocol and blood collection

Prior to the study day, subjects fasted overnight for 12 hours. They were instructed to take their normal medications and to drink plenty of water. The study was performed at the Clinical and Translational Science Center Clinical Research Center (CCRC) located at the VA Hospital in Mather, CA. On the study day, subjects arrived at the CCRC in the morning and were fitted with intravenous saline locks to obtain blood samples during the study. A fasting blood sample was collected, and then subjects were fed a standardized 40% saturated fat breakfast meal (Table 1). Following consumption of the breakfast meal, subjects were asked to rest quietly for the duration of the study day. Subjects were provided with *ad libitum* access to water, but were not allowed to consume any other foods or caloric beverages for the remainder of the study. Postprandial blood samples were obtained at 1.5, 3.5, and 6 hours after the breakfast meal. Blood samples were immediately placed on ice and processed within one hour for plasma isolation or flow cytometric studies. To isolate plasma, blood samples were drawn into K_2_EDTA Vacutainer tubes and centrifuged at 3,000 rpm for 15 minutes at 4°C. The plasma fraction was immediately frozen at −80°C until further analysis.

### Lipid analyses

#### Triglycerides and Cholesterol

Frozen/thawed plasma samples were used for all lipid analyses. Triglycerides, total cholesterol, and direct HDL cholesterol were determined colorimetrically after enzymatic hydrolysis and oxidation on a Poly-Chem instrument (PolyMedCo, Cortlandt Manor, NY) using the indicator quinoneimine. The Friedewald formula was used to calculate LDL cholesterol [28]: LDL cholesterol=total cholesterol−[HDL cholesterol+(triglyceride/5)].

#### Non-Esterified Fatty Acids

The non-esterified fatty acid (NEFA) concentrations of the plasma samples were measured in duplicate using an enzymatic colorimetric assay kit (Wako Diagnostics, Richmond, VA) according to the manufacturer’s instructions.

#### Oxylipin Analysis

The majority of circulating oxylipins are esterified into lipoprotein particles [29], and their release at the vascular endothelium by lipoprotein lipase may participate in inflammatory signaling [22]. Therefore sample extracts were subjected to alkaline hydrolysis prior to analysis to release this bound lipid pool, and a suite of arachidonic, eicosapentaenoic and docosahexaenonic acid metabolites were measured.

Frozen/thawed plasma samples were hydrolyzed and extracted by solid phase extraction (SPE) using 3 cc, 60 mg Oasis HLB columns (Waters, Milford, MA). Samples were enriched with an antioxidant solution of butylated hydroxyl toluene and the divalent cation chelator EDTA. Once eluted, samples were enriched with ~1 mg glycerol and residual solvent was removed under vacuum. Glycerol plugs were stored at 80°C until reconstitution in methanol-containing 100 nM of the internal standards 1-cyclohexyluriedo-3-dodecanoic acid (CUDA) and 1-phenyluriedo-3-hexanoic acid (PHAU).

Oxylipins and deuterated surrogates were analyzed by ultra-performance liquid chromatography-tandem mass spectrometry (UPLC-MS/MS) as previously described [30,31]. Briefly, analytes were separated on a 2.1×150 mm, 1.7 μm Acquity BEH column at 60°C on a Waters Acquity UPLC. Analytes were ionized in negative mode by electrospray ionization and data were acquired and collected in multi-reaction monitoring mode with an ABI 4000QTRAP triple quad mass spectrometer. Surrogate recoveries of reported analytes were acceptable.

### Metabolite analysis

Plasma samples for metabolomics assays were stored at −80°C and underwent two freeze-thaw cycles prior to extraction. The samples were extracted and derivatized as described previously [32]. The derivatized samples (0.5 μL) were injected onto an Agilent 6890 gas chromatograph (Santa Clara, CA) at 50°C (ramped to 250°C) in splitless mode with 25 seconds of splitless time. The chromatograph was equipped with a 30 m×0.25 mm i.d.×0.25 μm Rtx5Sil-MS column with a 10 m integrated guard column (Restek, Bellefonte, PA). The chromatography conditions were as follows: constant flow rate of 1 mL/min, oven temperature ramped from 50°C to 330°C, and total run time of 22 minutes. Mass spectrometry was accomplished by a Leco Pegasus IV time of flight mass spectrometer (St. Joseph, MI) under the following conditions: transfer line temperature of 280°C, electron ionization at −70eV, and ion source temperature of 250°C. Mass spectra were acquired from m/z 85–500 at 17 spectra s-1 and 1850 V detector voltage. All results were exported to the Fiehn Lab servers and processed by the metabolomics BinBase database [33,34]. All entries to the database were matched against both the Fiehn Lab mass spectral library of metabolites and the NIST05 commercial library using retention index and mass spectrum information. Quantifier ions were identified for each metabolite based on the information in BinBase. The quality control mixtures (containing 30 metabolites) established external 5-point calibration curves to control for instrument sensitivity. Furthermore, each individual chromatogram was controlled against the total number of identified metabolites and the total peak intensities in order to prevent outliers from confusing the statistical analysis. The differences in mean plasma metabolite concentrations between groups were evaluated using false discovery rate corrections.

### Cysteine and homocysteine measurements

Plasma homocysteine and cysteine were determined by high-pressure liquid chromatography with post-column fluorescence detection as described previously [35].

### Flow cytometry

Whole blood was drawn into sodium heparin Vacutainer tubes, immediately placed on ice, and aliquoted within 30 minutes for flow cytometry staining. An aliquot of whole blood was incubated with Brefeldin A (0.002%) for four hours at 37°C in a 95% air/5% CO2 incubator. Subsequent steps were performed at room temperature in the dark. Following the incubation period, small aliquots of the whole blood were stained with FITC-labeled mouse anti-CD14 antibody (catalog # 347493) for 20 minutes. Samples were lysed with FACS Lysing Solution, vortexed gently, and incubated for 10 minutes. Following centrifugation to collect the cells, samples were permeabilized using FACS Permeabilizing Solution for 10 minutes. Samples were washed once with wash buffer (PBS containing 0.5% BSA and 0.1% sodium azide) and then centrifuged to collect the cells. APC-labeled mouse anti-TNF-α (catalog # 340534) and PE-labeled mouse anti-IL-1β (catalog # 340516) antibodies were added to the samples and left to incubate for 30 minutes, after which they were washed once with wash buffer and fixed in 1% paraformaldehyde. Samples were run on a FACScan Flow Cytometer (BD Biosciences, San Jose, CA) and analyzed using FlowJo software (Tree Star, Ashland, OR). The monocyte population was determined by the light scatter characteristics of the cells, as well as the extracellular expression of CD14, a monocyte marker.

### Multiplex plasma cytokine analysis

Levels of plasma cytokines were measured using the Human ProInflammatory 7-Plex Assay Ultra-Sensitive Kit from Meso Scale Discovery (Gaithersburg, Maryland) according to the manufacturer’s instructions. The following cytokines were analyzed in duplicate: interleukin (IL)-1β, IL-12 p70, interferon-γ (IFN-γ), IL-6, IL-8, IL-10, and tumor necrosis factor (TNF)-α.

### Statistical analyses

All values are expressed as means ± standard deviation, unless otherwise noted. Normally-distributed data were analyzed using SigmaPlot software (San Jose, CA), and statistical significance was determined using two-way repeated measures ANOVA followed by the Holm-Sidak method for multiple comparisons testing, unless otherwise noted. A p-value of less than 0.05 was considered significant.

## Results

### Cholesterol and triglyceride levels are not different between AD and control groups during the fasting or postprandial states

Descriptions of the two study groups (Alzheimer’s patients and control subjects), including mean values for age, MMSE score, height, weight, and BMI are shown in Table 2. The differences between the two groups did not reach statistical significance for any of these parameters.

**Figure 1:**
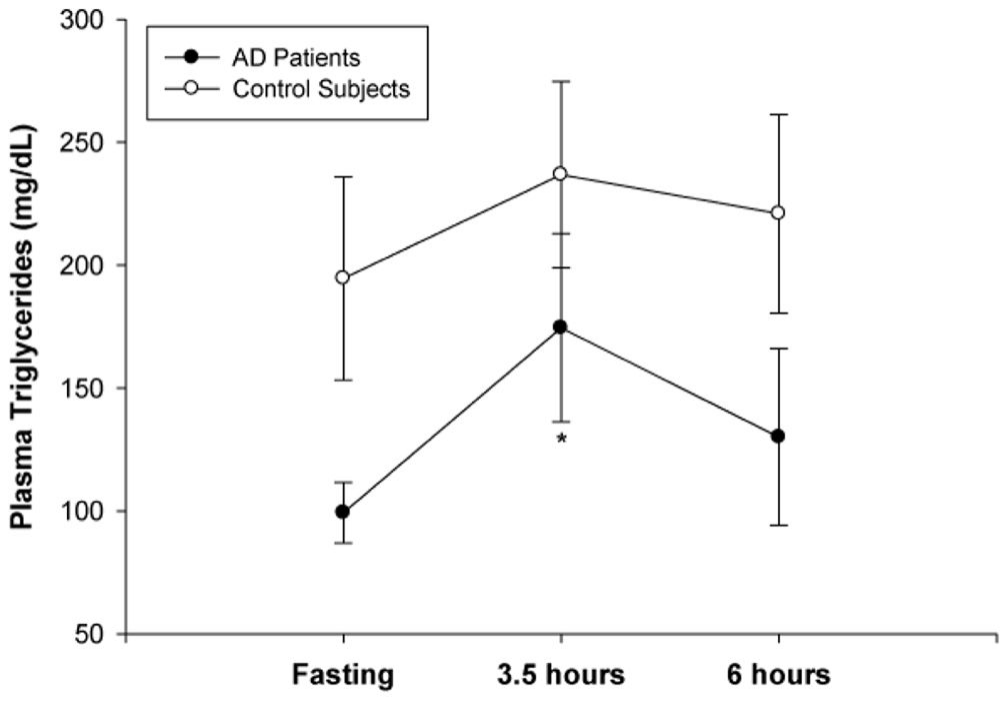
Plasma triglyceride levels in Alzheimer’s (AD) patients and control subjects. Whole blood samples were obtained at fasting, and at 3.5 and 6 hours following consumption of a moderately high-fat meal. The level of plasma triglycerides was not significantly different between the AD group (n=7) and the control group (n=9) at any time point. Both groups had increased levels of triglycerides at 3.5 hours postprandially, with this difference reaching significance in the AD group. Data are expressed as means ± SEM. ^*^p<0.05 compared to fasting (two-way repeated measures ANOVA).

One of our primary objectives was to characterize the postprandial lipid and lipoprotein response to a moderately high-fat meal challenge in AD patients compared to control subjects. To establish baseline values for the two groups, we first determined that fasting plasma cholesterol levels, including HDL, LDL, and total cholesterol, were not different in AD patients compared to control subjects (Table 2). As shown in Figure 1, plasma triglyceride levels were slightly higher in the control group compared to the AD group during fasting and the postprandial period, but these differences did not reach statistical significance. In agreement with previous studies [19,36], triglyceride levels increased from fasting to a postprandial peak at 3.5 hours, and approached baseline values at 6 hours.

### The relationship between postprandial NEFA and BMI differs between AD and control groups

**Figure 2:**
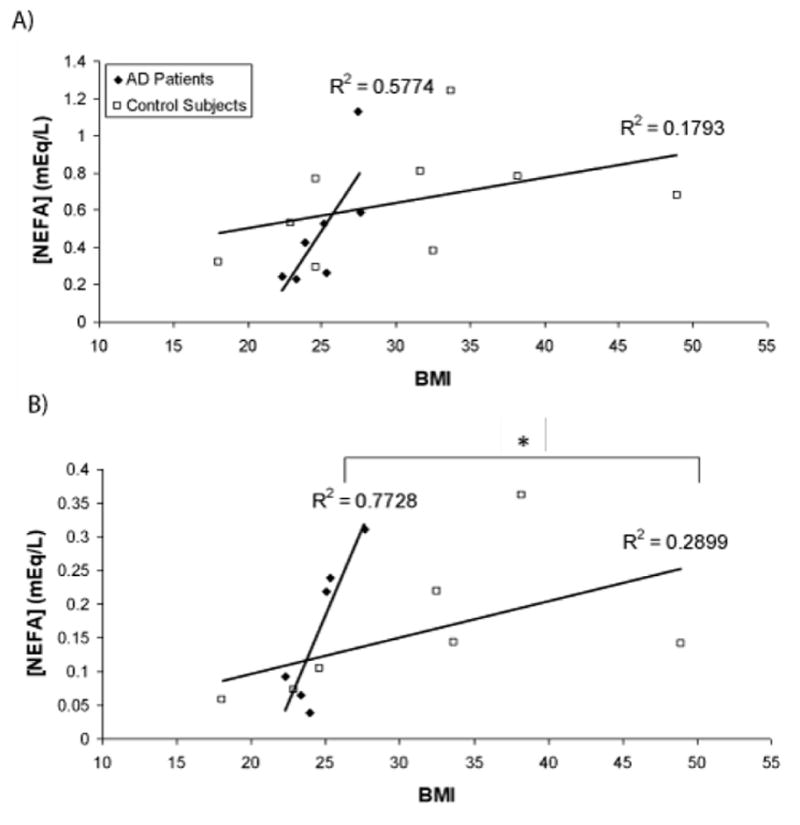
The relationship between non-esterified fatty acid (NEFA) levels and body mass index (BMI) differs between Alzheimer’s (AD) patients and control subjects. Plasma samples were collected during fasting (A) or 3.5 hours following consumption of a moderately high-fat meal (B). NEFA levels are expressed as a function of body mass index (BMI) for each subject. The slope of the relationship between plasma NEFA levels and BMI is significantly steeper in the AD group (n=6) compared to the control group (n=7) at the 3.5 hour time point (^*^p<0.05, ANCOVA). The linear relationship between plasma NEFA levels and BMI for each group is shown as a solid dark line, with the corresponding R2 value noted to describe the strength of the relationship.

As it is thought NEFAs help mediate postprandial inflammation, we investigated plasma concentrations of these lipids [22,23]. No differences in plasma NEFA were detected between the AD and control groups at any time point. The same trend was observed in each group, where NEFA concentration dropped from fasting during the postprandial period (through 3.5 hours) and approached baseline values at the 6-hour time point. When expressed as a function of BMI, however, the slopes of the linear relationship between BMI and NEFA levels were significantly different between the two groups at the 3.5-hour time point (Figure 2). This suggests that postprandial NEFA excursions are greater in AD patients relative to BMI-matched controls at 3.5 hours after the consumption of a moderately high-fat meal.

### Esterified eicosanoids and docosahexanoids do not differ between AD and control groups

**Figure 3:**
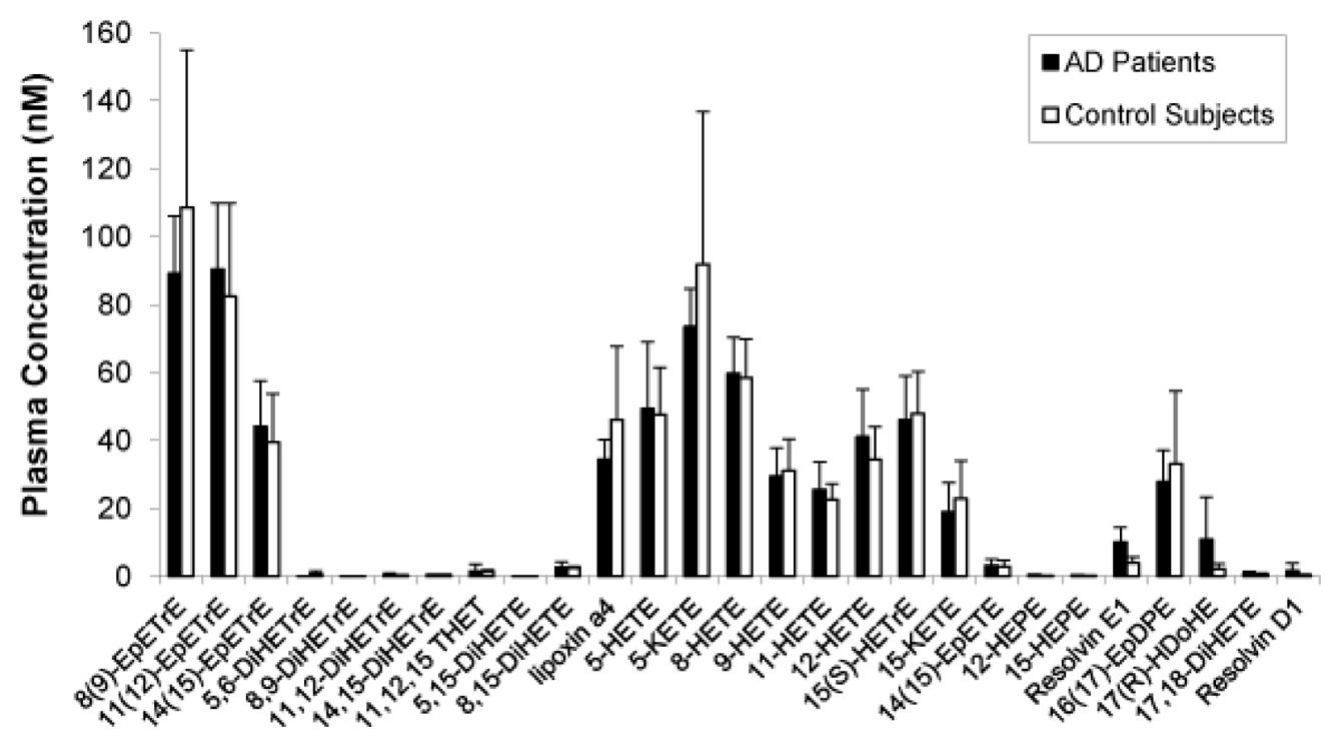
Oxylipin composition of plasma samples from Alzheimer’s (AD) patients and control subjects. Whole blood samples were obtained at fasting, after which the plasma fractions were isolated and analyzed by liquid chromatography-tandem mass spectrometry for the following analytes: 8(9)-epoxy-8Z,11Z,14Z-eicosatrienoic acid (EpETrE), 11(12)-EpETrE, 14(15)-EpETrE, 5,6-dihydroxy- 5Z, 8Z, 11Z- eicosatrienoic acid (DiHETrE), 8,9-DiHETrE, 11,12-DiHETrE, 14,15-DiHETrE, 11,12,15 trihydroxyeicosatrienoic acid (THET), 5,15-dihydroxy-5Z,9E,11Z, 13E-eicosatetraenoic acid (DiHETE), 8,15-DiHETE, lipoxin a4, 5-hydroxyeicosatetraenoic acid (HETE), 5- 5-oxo-6E,8Z,11Z,14Z-eicosatetraenoic acid (KETE), 8-HETE, 9-HETE, 11-HETE, 12-HETE, 15(S)-hydroxy-8Z,11Z,13E-eicosatrienoic acid (HETrE), 15-KETE, 14(15)-epoxy- 5Z, 8Z, 11Z, 17Z- eicosatetraenoic acid (EpETE), 12-hydroxy-5Z,8Z,10E,14Z,17Z-eicosapentaenoic acid (HEPE), 15-HEPE, Resolvin E1, 16(17)-epoxy-4Z,7Z,10Z,13Z,19Z-docosapentaenoic acid (EpDPE), 17(R)-hydroxy-4Z,7Z,10Z,13Z, 15E,19Z-docosahexaenoic acid (HDoHE), 17,18-dihydroxy-5Z,8Z, 11Z,14Z-eicosatetraenoic acid (DiHETE), and Resolvin D1. Data are expressed as means ± SD (n=5 AD patients, and 5 control subjects).

We then investigated the levels of plasma esterified oxylipins as an indication of oxidative stress [25,26]. Analytical performance was within acceptable limits for all reported analytes and the variance was equivalent between the two study groups. As shown in Figure 3, no significant differences were detected between control subjects and those with Alzheimer’s disease. Notably, the putative oxidative stress marker 9-HETE [37] was not significantly different between the subject groups, and isoprostanes were not routinely detected in these samples.

### Metabolomics analyses did not identify AD-specific changes in postprandial NEFA profiles

A metabolomics screen of plasma was performed to provide a broad assessment of metabolic changes in the cohorts. Of the 365 analytes observed in the screen, four in the fasting state and five in the postprandial state were different between groups; however, these differences did not reach statistical significance after controlling for false discovery rate (Table 3). The postprandial condition revealed elevated levels of behenic acid, cystine, and taurine, and reduced levels of linoleic and oleic acids in the Alzheimer’s group. During fasting, the AD patients had decreased levels of phosphoric acid and glyoxalurea, and elevated levels of fructose.

### No difference in fasting or postprandial homocysteine or cysteine levels between AD and control groups

As our initial metabolomics screen identified increased postprandial levels of the sulfur-containing amino acids cystine and taurine in the AD group, and previous studies have demonstrated a correlation between elevated plasma homocysteine (Hcy) levels and presence of Alzheimer’s disease [38–40], we further investigated plasma cysteine and Hcy levels. We conducted additional liquid chromatography measurements and demonstrated no changes in cysteine or Hcy levels between the two groups at any time point.

### Elevated levels of TNF-α-positive monocytes are present during fasting in AD patients, with no difference in IL-1β-positive monocytes between groups

**Figure 4:**
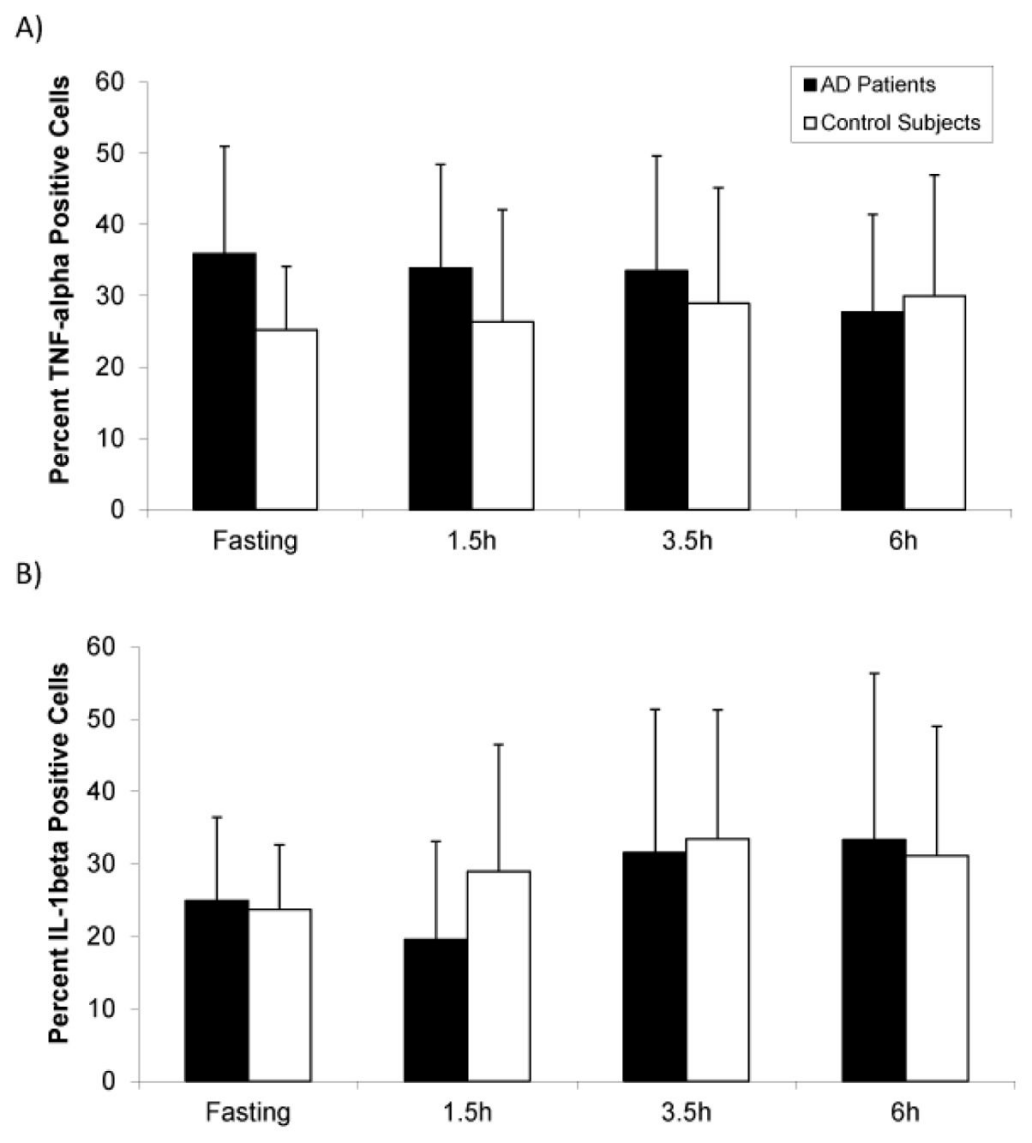
The percent of cluster of differentiation 14 (CD14)-positive monocytes expressing either intracellular tumor necrosis factor (TNF)-α (A) or intracellular interleukin (IL)-1β (B) in Alzheimer’s (AD) patients and control subjects. Whole blood samples were obtained from subjects during fasting, and then 1.5, 3.5, and 6 hours after consumption of a moderately high-fat meal and analyzed by flow cytometry. (A) There was a trend toward higher levels of TNF-α-positive monocytes in the AD group (n=6) compared to control (n=7). (B) There was no difference in the percent of IL-1β-positive monocytes between the AD (n=7) and control groups (n=8). Data are expressed as means ± SD, and p<0.05 was considered significant.

To investigate levels of vascular inflammation, we used flow cytometric analysis to determine the percent of monocytes expressing high levels of intracellular TNF-α or IL-1β. In the AD group, there was a higher percent of TNF-α-positive monocytes compared to the control group at the fasting, 1.5 hour, and 3.5 hour time points (Figure 4A). This increase was particularly prominent at the fasting time point, where the TNF-α-positive monocytes measured 35.9% in the AD group compared to 25.2% in the control group. However, this difference did not reach statistical significance. The percent of IL-1β-positive monocytes increased during the postprandial period, from fasting levels to a postprandial peak at 3.5 hours (Figure 4B). There was no difference in the percent of monocytes expressing IL-β between the AD and control groups at any time point.

### Increased levels of IL-10 and IL-12 p70 characterize the fasting state in AD patients

**Figure 5:**
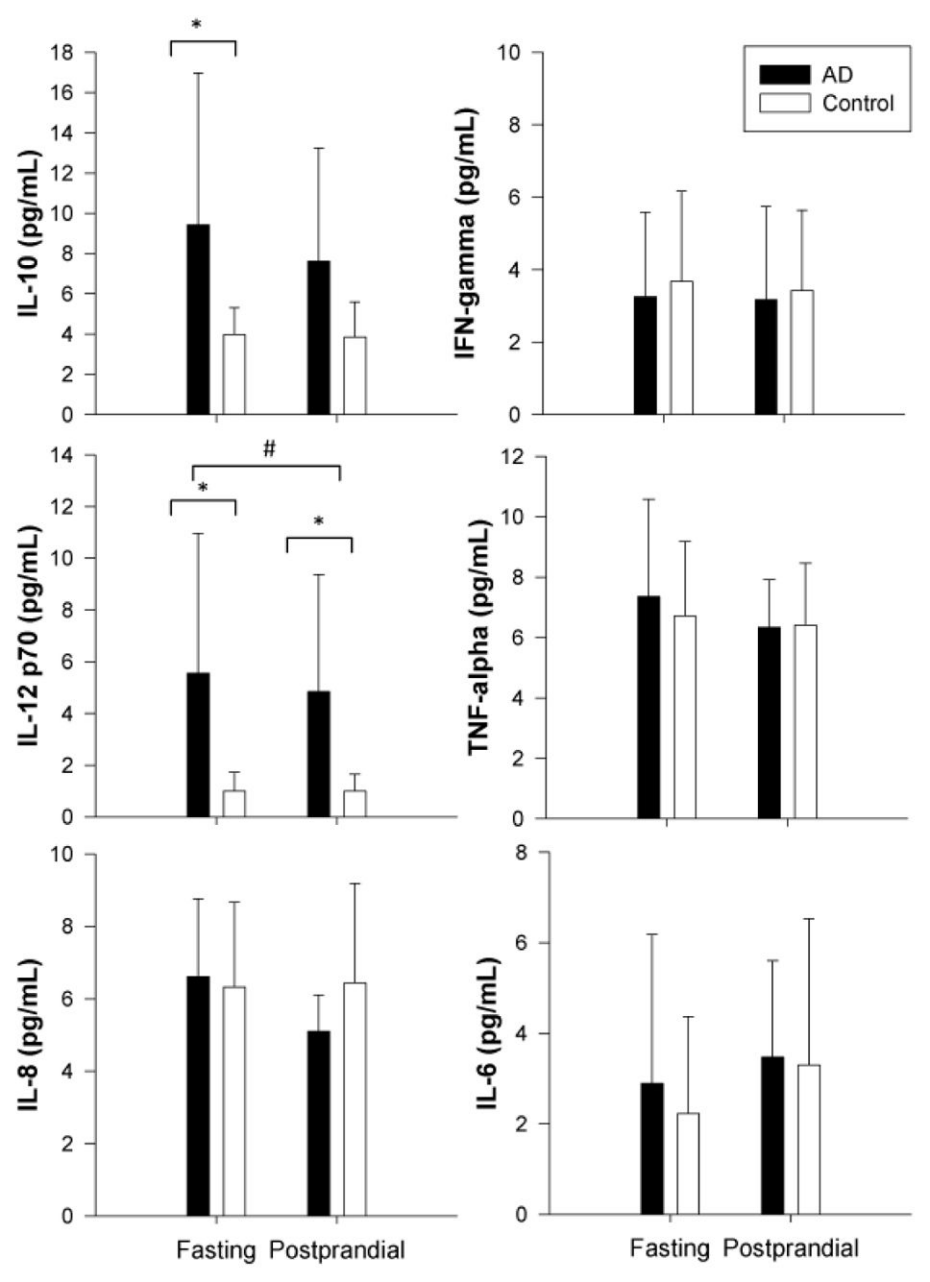
Plasma cytokine levels (pg/mL) in Alzheimer’s patients (AD) and control subjects (Control). Whole blood was obtained during fasting and 3.5 hours after consumption of a moderately high-fat meal (postprandial). The following cytokines were measured in the plasma fraction: interleukin (IL)-1β, IL-12 p70, interferon-γ (IFN-γ), IL-6, IL-8, IL-10, and tumor necrosis factor (TNF)-α. IL-10 and IL-12 p70 were elevated in AD patients during fasting, while IL-12 p70 was also elevated in AD patients in the postprandial period. Data are expressed as means ± SD. ^*^p<0.05 compared to control, and ^#^*p*<0.05 for fasting AD compared to postprandial AD (two-way repeated measures ANOVA).

We further sought to compare the vascular inflammatory state characteristic of the postprandial period between Alzheimer’s patients and control subjects through the multiplex analysis of a panel of cytokines classically related to inflammation. We assayed fasting and postprandial plasma samples for the following cytokines: interleukin (IL)-1β, IL-12 p70, interferon-γ (IFN-γ), IL-6, IL-8, IL-10, and tumor necrosis factor (TNF)-α. Our analysis revealed differences in IL-10 and IL-12 p70 between the AD and control groups. As seen in Figure 5, levels of both cytokines were increased in the Alzheimer’s patients during fasting compared to the control group, while IL-12 p70 was also elevated in the fasting state compared to the postprandial period in the AD group. The control group did not demonstrate a similar trend between time points.

## Discussion

Although previous studies have investigated the lipid profile and inflammatory markers in Alzheimer’s disease patients compared to cognitively normal subjects, to our knowledge, this is the first analysis of these same factors during the postprandial state. We hypothesized that consumption of a moderately high-fat meal is associated with elevated levels of potentially injurious lipids and exaggerated vascular inflammation in the postprandial period in AD patients. We demonstrate here the postprandial period following a moderately high-fat meal is not associated with an exaggerated inflammatory state in AD patients with minimal cognitive impairment compared to age-matched controls. However, we observed increased levels of activated monocytes, IL-10, and IL-12 p70 during fasting in AD patients. Our results also indicate the lipid profile of the AD group is characterized by reduced levels of linoleic and oleic acids, and an interaction between postprandial non-esterified fatty acid (NEFA) levels and body mass index.

It is increasingly recognized that inflammatory processes in the central nervous system characterize Alzheimer’s disease and may mediate neurotoxicity [41]. Some lines of evidence also suggest systemic and/or peripheral vascular inflammation may contribute to Alzheimer’s pathology [12,13]. Assessment of the fasting plasma oxylipin profiles in our study did not show evidence of a sustained elevation in lipoprotein oxidative damage in the AD patients, which would be consistent with a sustained oxidative stress, and we did not observe differences in 9-HETE, which has been reported as a marker of oxidative stress [37]. Furthermore, there were no significant differences in omega-6 and omega-3 polyunsaturated fatty acids, suggesting there was a similarity in background dietary lipids between the two groups [30]. However, these findings do not preclude changes in acute inflammatory reactions to a meal.

Of the inflammatory mediators investigated, we observed increased levels of IL-10 and IL-12 (p70 subunit) in the Alzheimer’s patients during the fasting state, however these differences were not sustained during the postprandial period. IL-10 and IL-12 play important roles in the differentiation and regulation of helper T (Th) cells as part of the cell-mediated immune response. The type I (Th1) response primarily functions in an inflammatory capacity and involves IL-12, while type II (Th2) cells serve as a major source of IL-10 that functions in an anti-inflammatory capacity [42–44]. It has been suggested that AD may in part reflect deficiency of a protective Th2 response to combat Th1-mediated pathology [45]. Our observations of elevations in fasting IL-10 suggest that further investigation into the balance between Th1 and Th2 responses in AD could be beneficial. Our results contrast those of previous studies that were unable to detect these two cytokines or observed no difference between serum and cerebrospinal fluid from AD and control groups [46–49]. However, it is important to note the majority of these studies did not specify the fasted or fed state of the subjects. We observed differences in IL-10 and IL-12 during the fasting state, suggesting the fasting environment may be more sensitive to the changes we detected.

Prior work has revealed the potential involvement of peripheral blood mononuclear cells (PBMCs) in inflammation in AD. Elevated levels of PBMCs from Alzheimer’s patients express pro-inflammatory TNF-α and cyclooxygenase (COX)-2, pro-apoptotic PARP-1, and the adhesion-related marker of activation, CD38. [11] We observed a non-significant trend towards a higher percentage of TNF-α-positive monocytes in the Alzheimer’s group at fasting consistent with these previous studies.

It is interesting to note that the greatest increase in TNF-α-positive monocytes in the AD group coincided with the point at which total plasma non-esterified fatty acid (NEFA) levels were highest, i.e. the fasting state. This observation may potentially be indicative of higher vascular inflammatory activity at similar concentrations of NEFAs. Futhermore, we observed a correlation between NEFA levels and BMI that was significantly stronger in the Alzheimer’s group 3.5 hours postprandially. Relative to their BMI, AD subjects had higher levels of NEFAs, which invites the question of whether they have a greater propensity for inflammatory activation. Further studies are needed to elucidate the underlying mechanism behind this observation and to account for potential effects of gender that might influence BMI, as well as adipose distribution and lipolytic behavior.

After subjects consumed a 40% saturated fat meal, we noted an increasing trend in the plasma levels of triglycerides from fasting to a postprandial peak at 3.5 hours. This correlates well with previous studies showing a similar peak in plasma triglycerides at about 3.5 hours after subjects consumed a moderately high-fat meal. [19] The postprandial increase in triglcyerides was more pronounced in the AD group than the control group. However, the control group showed consistently higher levels of plasma triglycerides at each time point (fasting, 3.5 hours and 6 hours postprandially,) which may help account for the blunted postprandial response. Measurements of plasma triglyceride levels in AD patients compared to control subjects have met with considerable variation among studies. Our observation is consistent with reports of decreased triglyceride levels in AD patients with a similar degree of dementia compared to our patients (as evidenced by the MMSE scores) [50].

To further investigate the distribution of specific fatty acids across time points between the two groups we conducted a global metabolomics analysis. This experiment revealed differences in the plasma concentrations of three fatty acids in the postprandial period: behenic acid levels were elevated, while oleic and linoleic acids were decreased in the AD group. These results correlate well with previous studies demonstrating reduced plasma and brain levels of oleic and linoleic acids in AD patients [51–53]. Whether the observed alterations in these two fatty acids reflect differences in intake or metabolism, or a combination of the two, presents an intriguing question. It would be beneficial to provide a controlled dietary regimen prior to the defined study breakfast meal to address this question. We further probed the sulfur-containing amino acids cysteine and homocysteine as part of our metabolomics analysis and did not observe any differences between the two study groups. However, this does not preclude the possibility that subtle changes in sulfur metabolism may be occurring in the postprandial period in AD subjects, and these changes may have direct relevance to the reported dysregulation of cysteine/homocysteine in Alzheimer’s disease [38–40,54].

The primary aspect of our study design warranting further consideration is the small number of subjects. However, other investigations focused on postprandial inflammatory responses following various dietary challenges used similarly small samples sizes [55–58]. In our cohort each subject served as their own control, which helped reduce variability among the different time points. For vulnerable populations, such as cognitively impaired Alzheimer’s patients, the feasibility of recruiting large numbers of individuals is questionable. The primary aim of the studies presented here was to establish preliminary observations regarding the postprandial response to a moderately high-fat diet challenge in AD. Large cohorts of study subjects were beyond the scope of this pilot project and are planned for future work based on our initial pilot observations.

Finally, the evolution of AD ranges from the preclinical with no cognitive impairment to the vegetative state. The quantity and composition of food intake also varies considerably during this evolution, which would be expected to have effects on the postprandial inflammatory response. We have focused our study on those AD patients with minimal cognitive impairment with the hope that intervention at this early stage of the disease could attenuate the disease process. As biomarkers of AD improve, future studies should investigate the postprandial state in those persons at high risk for developing cognitive impairment.

In conclusion, we observed a combination of increased pro- and anti-inflammatory mediators, including IL-10, IL-12, and TNF-α-positive monocytes in Alzheimer’s patients during fasting, and a stronger interaction between postprandial NEFA levels and body mass index in the AD group compared to the control subjects. This may be further evidence of a heightened inflammatory state in AD. However, our results do not suggest that this inflammation is exacerbated in the postprandial state. We also identified differences in the metabolomics profile between AD and control subjects, which suggest potential targets for future investigations. Our work has clinical relevance in that it provides insight into potential baseline inflammatory mechanisms and metabolic differences that may characterize Alzheimer’s disease.

## Figures and Tables

**Table 1:** Composition of the standardized breakfast meal containing approximately 40% of total energy derived from fat. Nutrient content was determined using First DataBank Nutritionist Pro, version 2.0.90, 2004.

Food Item	Amount (g)	Energy (kcal)	Protein (g)	Carbohydrate (g)	Fat (g)
Plain Bagel, 1 small	75.0	206.3	7.9	40.1	1.2
Cream Cheese, 4 tsp	19.3	67.5	1.5	0.5	6.7
Egg, 1 large	50.0	74.5	6.2	0.6	5.0
Margarine, 2 tsp	9.4	67.6	0.1	0.1	7.6
Cantaloupe, 1 cup	226.8	79.4	2.0	19.0	0.6
Whole Milk, 1 cup	244.0	148.8	8.0	11.4	8.2
**Total**	624.5	644.0	25.7	71.6	29.3
**% of energy**			15.7	43.9	40.4

**Table 2:** Characteristics of Alzheimer’s patients and control subjects enrolled in the study.

	AD Patients	Control Subjects
No. of individuals (M/F)	7(3/4)	9 (3/6)
MMSE	22.7 ± 2.4	>26
Age, years	78 ± 9.3	76 ± 6.8
Height, cm	162.7 ± 9.44	165.9 ± 8.13
Weight, kg	66.2 ± 8.14	86.0 ± 32.7
BMI, kg/m2	25.0 ± 2.00	30.6 ± 9.34
Total cholesterol, mg/dL	222.6 ± 48.3	220.7 ± 42.3
HDL, mg/dL	61.9 ± 16.7	54.8 ± 15.1
LDL, md/dL	140.8 ± 27.8	127.0 ± 37.5

MMSE=Mini-Mental State Exam [27]; HDL = high density lipoprotein; LDL = low density lipoprotein. Clinical lipid measurements were obtained during fasting. Data are expressed as means ± SD. The differences between the two groups did not reach statistical significance (p<0.05)

**Table 3:** Metabolites displaying trends toward different plasma concentrations in Alzheimer’s patients (AD) and control subjects*.

	AD	Control	*P*-value
**Postprandial**			
Behenic (docosanoic) acid	959 ± 116	759 ± 234	0.046
Linoleic acid	619 ± 218	925 ± 273	0.026
Oleic acid	1597 ± 683	2577 ± 1124	0.05
Cystine	14343 ± 2768	10214 ± 4575	0.043
Taurine	846 ± 328	461 ± 362	0.044
**Fasting**			
Phosphoric acid	74528 ± 8966	88939 ± 8020	0.0058
Glyoxalurea	1555 ± 281	1900 ± 254	0.025
Fructose	2215 ± 670	1471 ± 408	0.029

* Data are expressed as means ± SD. *P*-values determined by t-test and reported for those metabolites with *p* ≤ 0.05 compared to control. After controlling for false discovery rate, none of the metabolites were shown to be significantly different between groups.
